# Nurses’ educational needs in the oral health of inpatients at Yazd Province in Iran: a Delphi study

**DOI:** 10.1186/s12912-020-00517-8

**Published:** 2020-12-11

**Authors:** Seyed Hosein Tabatabaei, Fatemeh Owlia, Fatemeh Ayatollahi, Fahimeh Rashidi Maybodi, Hakimeh Ahadian, Fatemeh Azizian, Khadijeh Nasiriani

**Affiliations:** 1 Department of Oral and Maxillofacial Pathology, Social Determinants of Oral Health Research Center, School of Dentistry, Shahid Sadoughi University of Medical Sciences, Yazd, Iran; 2 Department of Oral and Maxillofacial Medicine, School of Dentistry, Shahid Sadoughi University of Medical Sciences, Yazd, Iran; 3 Endodontics Department, School of Dentistry, Shahid Sadoughi University of Medical Sciences, Yazd, Iran; 4 Department of Periodontics, School of Dentistry, Shahid Sadoughi University of Medical Sciences, Yazd, Iran; 5 Medical Education, School of Dentistry, Shahid Sadoughi University of Medical Sciences, Yazd, Iran; 6 Department of Nursing, School of Nursing, Mother and Newborn Health Research Center, Nursing and midwifery Research Center, Shahid Sadoughi University of Medical Sciences, Yazd, Iran

**Keywords:** Oral hygiene, Oral health, Patient, Needs assessment, Nurse, In-patients

## Abstract

**Background:**

Oral hygiene is an integral part of general health of a person. Nurses qualified about oral care can play an important role in improving the quality of oral health in hospitalized patients. This study investigated the educational needs of nurses in the field of oral health of hospitalized patients.

**Methods:**

The study used the modified Delphi method in three rounds. Fifty faculty members of the School of Dentistry and Nursing were selected via purposive sampling. The data collection tool was a demographic form and an open-ended questionnaire in the first round and a structured questionnaire in the next rounds. The analysis was performed using both content and descriptive analysis techniques.

**Results:**

The top ten oral health education priorities for nurses were greater than 75% with a consensus level: oral anatomy and physiology, learning the signs and symptoms of common oral diseases, learning of oral medications and administration, learning the drugs that cause damage to the mouth and teeth, training in managing dental emergencies, patient education for tooth brushing and taking care of the mouth, especially in the elderly patient, providing oral and dental care, training for unconscious and fasting patients, undergoing chemotherapy and radiotherapy, and hospitalization in intensive care unit.

**Conclusion:**

The findings of the study emphasized the need for interdisciplinary cooperation between nursing and dental professionals for the development of an oral health curriculum for nurses to promote and improve oral health and prevent dental diseases in hospitalized patients and the community.

**Supplementary Information:**

The online version contains supplementary material available at 10.1186/s12912-020-00517-8.

## Background

Oral hygiene is an integral part of general health that should be maintained throughout life, as the mouth is considered the mirror of the body and the gateway to health [1, 2]. Numerous studies have shown the poor condition of oral hygiene and inadequate access to oral care in hospitalized patients [3] and that oral health (OH) deteriorates during hospitalization [4]. OH is neglected, especially in the elderly, partly because they need to care on many levels; as a result, less time is reserved for OH care [5]. Also, plaque and tongue coverage indices increase with hospital stay in the intensive care unit [6]. In the other words, hospitalization is associated with deterioration of OH, particularly in intubated and elderly patients [7, 8].

The study by Carrilho Neto et al. (2011) showed most of the patients had poor oral hygiene, gingivitis, periodontal disease, and dental decay [9]. This has been due to the lack of promotion and control program in the OH care setting [10]. On the other hand, various studies have confirmed the effects of oral diseases, especially periodontal diseases, on some systemic diseases including cardiovascular disease [11], lung disease (including COPD) and pregnancy problems and complications, including low birth weight, premature birth, growth retardation, and preeclampsia [12]. Most of oral diseases can be prevented [13].

Nurses are one of the largest healthcare group [14] that play an important role in providing health care services and promoting health and disseminating preventive information [13, 15]. Nurses may be important in detecting oral diseases and educating patients, especially in countries with limited human resources and a shortage of dental professionals [16, 17]; Yao et al. (2019) & Philip et al. (2019) showed the nurses’ knowledge was poor and inadequate in OH care [1, 15]. Also, oral care is insufficient in hospitalized patients, especially high-risk patients. This could be caused by lack of training [18, 19].

In general, studies have shown that nurses need to improve their knowledge about OH [13, 20]. Therefore, more attention should be paid to the issue of OH in nursing curriculum [13, 19, 20]. Considering the importance of OH for the promotion and maintenance of public health and welfare [14, 20], it is so necessary to maintain OH in people hospitalized to improve wellbeing outcomes and quality of life [21]. Besides, the negative consequences of poor OH in hospitalized patients are significant, specially in patients with an artificial airway [14]. Nurses, as primary care providers, need sufficient knowledge, positive attitude, and acceptable performance to be able to provide effective OH care [21]. This issue should be considered in nurses’ training programs. Nonetheless, studies show an obvious lack in nursing curriculum [14, 20]. In a national survey of 1000 US nursing education programs, more than half of schools reported that there was a shortage of curriculum content, and lack of faculty time, interest, and expertise in health education, including OH [14]. Likewise, Jablonski (2012) demonstrated the content devoted to oral hygiene in nursing textbooks averaged 0.6% [22]. Based on the above-mentioned issues, this study was designed to investigate the educational needs of nurses about the OH of the hospitalized patients.

## Methods

This study was performed using the modified Delphi method over three rounds. The Delphi method is a forecasting process framework based on the results of multiple rounds of questionnaires sent to a panel of experts that seeks to reach a correct response through consensus on a particular topic [23, 24]. The panelist requires several characteristics such as: knowledge and experience in the subject, willingness and enough time to participate. The panelists of this study were faculty members and instructors of dentistry school and nursing school working at Shahid Sadoughi University of Medical Sciences, Yazd Province, Iran. The panelists were selected by purposive sampling. Participating nurses had at least 2 years of clinical work experience in the hospital and dentists also had work experience in the field of OH of hospitalized patients. The number of participants in Delphi’s research is usually less than 50 and mostly 15 to 20 [25]. In this study, considering that two groups of nursing and dentistry participated and with the probability of attrition of panelists in the rounds, 25 samples were considered in each group and a total of 50 people participated.

Data collection tool was a questionnaire. In each round, a summary of the results of the previous round was evaluated by the panelists. In the first round, an unstructured questionnaire was sent to the participants by e-mail, and they were requested to complete the demographic information including age, gender, level of education, work experience and an open-ended question: “In your opinion, what are the educational priorities of nurses in the field of improving the OH of hospitalized patients?” Participants were free to express any ideas and opinions about the educational needs of OH nurses and write a list of topics of interest. The items obtained from the first round were analyzed. Also, at the end of this round, the literature was reviewed for finding related issue, the categories extracted from the first round as a structured questionnaire to be used as tools for the second round. In the second round, the participants were asked to answer the items on a Likert scale (not important, important, and very important). Therefore, about the importance of ranking, only items were accepted that scored above 75% and were ranked as important and very important. In the third round, all items that were accepted in the second round were sent to the experts to express their agreement or disagreement with the items. The items were accepted that obtained an agreement score above or equal to 75%. In most Delphi studies, the criterion for consensus was percentage of agreement by 75% [26]. The remained approved items were declared as OH educational needs required by nurses.

The analysis was performed using both quantitative and qualitative methods. Data analysis was performed in the first round of this study with conventional content analysis approach [27]. Qualitative content analysis is one of the qualitative methods currently available for analyzing data and interpreting its meaning and an autonomous method that can be used at varying levels of abstraction and interpretation [28]**.** In conventional content analysis, coding categories are derived directly from the text data. In this study, conventional content analysis was done based on the Lundman & Graneheim method in which codes, category, and themes are compared for similarity and differences [29]. IBM SPSS Statistics 16 was used to analyze the absolute and relative frequency in the second and third rounds.

This study was the second phase of a project of the designing, implementation and evaluation of a specific nursing educational program in OH of hospitalized patients, which was approved by the Committee of Ethics in Human Research at Shahid Sadoughi University of Medical Sciences in Yazd. In this study, an informed consent was obtained from the participants. Eligible participants were informed about the aim of the study, the method, and confidentiality of their responses by e-mail.

## Results

In this study, 25 nurses and 25 dentists participated in the first round. (The demographic characteristics are presented in Table 1). In the second round, 2 dental faculty members did not return the questionnaire. Also, in the third round, 1 nursing faculty member did not return the questionnaire.

**Table 1:** Demographic characteristics of the participants

Variables	Data value
Gender	
Female	37 (74)
Male	13 (26)
Field of Study	
Nursing	25 (50)
Dentistry	25 (50)
Degree of Education	
M.Sc. degree	17 (34)
PhD or professional doctorate degree	33 (66)
Age (yr)	34.9 ± 7.36
work experience (yr)	7.94 ± 6.75

Values are given as n(%) or range (mean)

In the first round, the participants were asked to write the educational needs of nurses in the OH of inpatients. The dental participants further stated aspects of OH theory such as oral anatomy and physiology, familiarity with the signs and symptoms of common oral diseases, etc. However, the nurses emphasized more the clinical aspects of oral care such as providing oral for NPO and the elderly patient, etc. The results of the open questionnaires were analyzed by content analysis, which resulted in the extraction of 300 initial codes which were placed on the first 27 categories (Table 2).

**Table 2:** Primary categories extracted in the qualitative content analysis of the first round

≠	*Primary Categories*	Number of codes
1	Oral Anatomy and physiology	24
2	Familiarity with the signs and symptoms of common oral diseases	20
3	The relationship between diseases such as respiratory diseases, diabetes and oral health problem	10
4	Teaching dental emergencies (Sudden toothache, cheek swelling due to toothache.)	18
5	Introducing drugs that affect oral disorders (methods of treating oral problems, such as in radiotherapy)	18
6	Education of periodic examination of the mouth	10
7	Problems with wearing dentures and how to remove them	8
8	Familiarity with prescribed medications for oral problems and oral medication administration	25
9	Introducing drugs that cause damage to the mouth and teeth	36
10	Familiarity with interventions controlling bad breath (halitosis)	5
11	How to use dental consultation and refer to a dentist	11
12	Teaching children periodic oral examinations	5
13	Mouthwash training	12
14	Brushing training for special patients	30
15	Patient education for tooth brushing and taking care of the mouth, especially in the elderly patient	40
16	Teeth fluoride varnish training	5
17	Providing oral and dental care for unconscious patients	30
18	Providing oral and dental care for NPO patients	32
19	Providing oral and dental care for patients undergoing chemotherapy and radiotherapy	22
20	Providing oral and dental care for patients under *oropharyngeal candidiasis*	8
21	Providing oral and dental care for patients with maxillofacial trauma	13
22	Providing oral and dental care for patients admitted to critical care unit	41
23	Education of proper nutrition	10
24	Electric toothbrush	3
25	Flossing	6
26	Community health education and oral and dental care	5
27	Providing oral and dental care for pregnant mothers	2

After extracting the presented categories from the interview, the literature was reviewed. Then, the categories were revised and 20 items were sent for the second round in a structured questionnaire. In the second round, the importance of the items was examined. It was considered an accepted item if the sum of the relative frequency level of importance (Very Important and Important) was above 75% and the rest were removed. The removed 6 items were: introducing drugs that affect oral disorders (methods of treating oral problems, such as in radiotherapy), how to use dental consultation and refer to a dentist, teaching children periodic oral examinations, brushing training for special patients, and teeth fluoride varnish training. In the third round, the items approved in the second round were sent to experts in the agreed/disagreed format to prioritize nurses’ educational needs in the OH of in-patients. Items for which the panelists reached an agreement above 75% were accepted for the last round and the rest were eliminated. So, 5 items on “the relationship between diseases such as respiratory diseases, diabetes, heart disease, etc. and OH problem”, “problems with wearing dentures and how to remove them”, “familiarity with interventions for controlling halitosis (bad breath)”, “providing oral and dental care for patients under *oropharyngeal candidiasis”,* and “providing oral and dental care for patients with maxillofacial trauma” were removed. (Table 3).

**Table 3:** Results of the Second & Third Round Delphi

≠	Item of educational needs of nurses in oral care of hospitalized patients	Important			Consensus	
		Very important	Important	Not important	Agree	Disagree
		n(%)	n(%)	n(%)	n(%)	n(%)
1	Oral Anatomy and physiology	15 (30%)	32 (64%)	1 (2%)	41 (87%)	6 (13%)
2	Learning the signs and symptoms of common oral diseases	14 (29%)	29 (60.5%)	5 (10.5%)	42 (89%)	5 (11%)
3	The relationship between diseases such as respiratory diseases, diabetes, heart disease, etc. and oral health problem	11 (23%)	31 (64.5%)	6 (12.5%)	8 (17%)	39 (83%)
4	Training in managing dental emergencies (Sudden toothache, cheek swelling due to toothache, etc.).	6 (12.5%)	27 (56.25%)	15 (30.25%)	36 (77%)	11 (23%)
5	Learning the drugs that affect oral disorders (methods of treating oral problems, such as in radiotherapy)	5 (10.5%)	12 (25%)	31 (64.5%)		
6	Problems with wearing dentures and how to remove them	10 (21%)	33 (68.5%)	5 (10.5%)	11 (23%)	36 (77%)
7	Leaning the oral medications and oral medication administration	7 (15%)	34 (75%)	7 (15%)	39 (83%)	8 (17%)
8	Learning drugs that cause damage to the mouth and teeth	8 (17%)	38 (79%)	2 (4%)	36 (78%)	11 (22%)
9	Familiarity with interventions for controlling halitosis (bad breath)	8 (17%)	33 (68.5%)	7 (14.5%)	5 (11%)	42 (89%)
10	How to use dental consultation and refer to a dentist	15 (31.25%)	11 (23%)	22 (45.75%)		
11	Teaching children periodic oral examinations	12 (25%)	10 (21%)	26 (54%)		
12	Brushing training for special patients	2 (4%)	18 (37.5%)	28 (58.5%)		
13	Patient education for tooth brushing and taking care of the mouth, especially in the elderly patient	18 (37.5%)	29 (60.5%)	1 (2%)	98 (96%)	1 (2%)
14	Teeth fluoride varnish training	10 (21%)	5 (10.5%)	33 (68.5%)		
15	Providing oral and dental care for unconscious patients	36 (75%)	8 (17%)	4 (8%)	38 (81%)	9 (19%)
16	Providing oral and dental care for NPO patients	34 (70.5%)	8 (17%)	6 (12.5%)	40 (85%)	7 (15%)
17	Providing oral and dental care for patients undergoing chemotherapy and radiotherapy	41 (85.5%)	5 (10.5%)	2 (4%)	40 (85%)	7 (15%)
18	Providing oral and dental care for patients under *oropharyngeal candidiasis*	38 (79%)	9 (19%)	1 (2%)	11 (22%)	36 (78%)
19	Providing oral and dental care for patients with maxillofacial trauma	37 (77%)	10 (21%)	1 (2%)	7 (15%)	40 (85%)
20	Providing oral and dental care for patients admitted to critical care unit	41 (85.5%)	6 (12.5%)	1 (2%)	98 (96%)	1 (2%)

Finally, the 10 approved items were confirmed as educational priorities for OH in nursing (Table 4).

**Table 4:** Oral health nursing educational needs in hospitalized patients

≠	Oral health education priorities for nurses
1	Oral Anatomy and physiology
2	Learning the signs and symptoms of common oral diseases
3	Learning of oral medications and *administration*
4	Learning the drugs that cause damage to the mouth and teeth
5	Training in managing dental emergencies
6	Patient education for tooth brushing and taking care of the mouth, especially in the elderly patient
7	Providing oral and dental care training for unconscious patients
8	Providing oral and dental care training for NPO patients
9	Providing oral and dental care for patients undergoing chemotherapy and radiotherapy
10	Providing oral and dental care for patients admitted to critical care unit

## Discussion

In this study, the educational needs of nurses in oral and dental health of hospitalized patients were identified. According to some studies, nurses are not satisfied with the health of the mouth and there is a need for training [30, 31]. Therefore, there is a need for training and education.

According to the findings, there were ten educational priorities of the nurses in the field of OH for hospitalized patients. Of course, each has been a topic and the educational content must be developed for each of them.

Based on the findings, nurses who want to provide oral care for their patients in hospital must know the oral anatomy and physiology and the signs and symptoms of common oral diseases. They should have enough information about the medications that are prescribed to fight oral and dental diseases or about the medicines which can cause dry mouth (xerostomia) and increase the risk of tooth decay and drug administration route. Nurses need to learn what to do when a patient has tooth pain or swollen gums due to toothache.

Based on important findings of the study, that nurses need to learn about delivery of oral care to hospitalized patients is that the elderly are more often hospitalized and with longer hospital stays compared to other age groups; so, nurses must learn how to brush their teeth and take care of the mouth, especially in the elderly patient. Haresaku et al. (2018) showed almost half the nurses performed OH check-ups for elderly patients [32].

Another important finding is that nurses need to learn is concerned with delivery of oral care to hospitalized patients. It was emphasized that nurses should be able to provide OH care in this particular group of patients, because they form a greater number of patients requiring hospitalization. These groups of patients include NPO and unconscious, admitted to critical care unit and undergoing chemotherapy and radiotherapy, critically ill patients with an artificial airway. Lee et al. (2019) indicated most participants considered that there is a need for evidence-based protocol for oral care for hospitalized patients, and nurses employed in the critical care unit are much more responsible in this regard compared to nurses working in the public ward [33].

Review of the related literature showed insufficient research to determine the educational requirements of OH for nurses, especially hospitalized patients. Moreover, the studies have been conducted about basic OH. Rwakatema et al.(2015) declared nursing students had insufficient knowledge about basic oral hygiene [34]. However, studies showed positive impact of educational programs in OH. Khanbodaghi et al. (2019) suggested the inter-professional education program in oral hygiene issues that can help ongoing dental surveillance [35].

On the basis of findings, the nurse requires more attention to assess the status of OH and offer the related care. In particular, mouth/oral care should be done for elderly, high-risk patients, and in hospitalized patients in Intensive Care Unit. In this regard, interdisciplinary cooperation between nurses and dentists must be enhanced to develop an OH care curriculum. Then, nurses should be educated about in-patients’ oral care needs. In the future, well-qualified nursing workers can promote OH and prevent dental disease in hospitalized patients.

One of the limitations of the present study was the lack of participation of nursing and dental specialists from other universities and throughout the country. It is suggested that this shortcoming be considered in future studies.

## Conclusion

In this study, ten topics were noted as the most important educational needs of nurses in the field of OH care. The educational needs assessment of nurses about OH of in-patients can lead to the designing, implementation and evaluation of the special nursing OH curriculum for training of undergraduate and graduate courses and nurses retraining.

## Supplementary Information

**Additional file 1.** Delphi First Round Questionnaire

**Additional file 2.** Delphi Second Round Questionnaire

**Additional file 3.** Delphi Third round questionnaire

## Data Availability

The datasets used and/or analyzed during the current study are available from the corresponding author on reasonable request.

## Abbreviations

COPD: Chronic obstructive pulmonary disease
OH: Oral health
NPO: *Nil* per os = nothing by mouth
