# Supplementary material for: Nurses’ educational needs in the oral health of inpatients at Yazd Province in Iran: a Delphi study
Source: BMC Nurs. 2020 Dec 11;19:120. doi: 10.1186/s12912-020-00517-8 (PMC7733290; doi:10.1186/s12912-020-00517-8)
Supplement: Supplementary file 2 — Additional file 2. Delphi Second Round Questionnaire [file 12912_2020_517_MOESM2_ESM.docx]

**Delphi Second Round Questionnaire**

Dear Colleague
Greetings and good health

Thank you very much for your valuable comments in the first round of Delphi, the study "**Nurses' educational needs in the oral health of inpatients: Delphi** method ". Researchers declare, your comments in first round analyzed. Also, the topics related to the subject have been extracted from books and articles. The structured questionnaire has developed for Collection of data in second round. At this round, you will be asked again to evaluate the importance of the results obtained and record your answers. Thank you for patiently accompanying us in this study.

| ≠ | Oral health education priorities for nurses | very important | important | Not important |
| --- | --- | --- | --- | --- |
| 1 | Oral Anatomy and physiology |  |  |  |
| 2 | Learning the signs and symptoms of common oral diseases |  |  |  |
| 3 | The relationship between diseases such as respiratory diseases, diabetes, heart disease, etc. and oral health problem |  |  |  |
| 4 | Training in managing dental emergencies (Sudden toothache, cheek swelling due to toothache, etc.). |  |  |  |
| 5 | Learning the drugs that affect oral disorders (methods of treating oral problems, for example in radiotherapy) |  |  |  |
| 6 | Problems with the use of dentures and how to remove them |  |  |  |
| 7 | Leaning the oral medications and oral medication administration |  |  |  |
| 8 | Learning drugs that cause damage to the mouth and teeth |  |  |  |
| 9 | Familiarity with interventions for controlling halitosis (bad breath) |  |  |  |
| 10 | How to use dental consultation and refer to a dentist |  |  |  |
| 11 | Teaching children periodic oral examinations |  |  |  |
| 12 | Brushing training for special patients |  |  |  |
| 13 | Patient education for tooth brushing and taking care of the mouth, especially in the elderly patient |  |  |  |
| 14 | Teeth fluoride varnish training |  |  |  |
| 15 | Providing oral and dental care for unconscious patients |  |  |  |
| 16 | Providing oral and dental care for NPO patients |  |  |  |
| 17 | Providing oral and dental care for patients undergoing chemotherapy and radiotherapy |  |  |  |
| 18 | Providing oral and dental care for patients under oropharyngeal candidiasis |  |  |  |
| 19 | Providing oral and dental care for patients with maxillofacial trauma |  |  |  |
| 20 | Providing oral and dental care for patients admitted to critical care unit |  |  |  |
