# Supplementary material for: Nurses’ educational needs in the oral health of inpatients at Yazd Province in Iran: a Delphi study
Source: BMC Nurs. 2020 Dec 11;19:120. doi: 10.1186/s12912-020-00517-8 (PMC7733290; doi:10.1186/s12912-020-00517-8)
Supplement: Supplementary file 3 — Additional file 3. Delphi Third round questionnaire [file 12912_2020_517_MOESM3_ESM.docx]

**Delphi Third round questionnaire**

Dear Colleague
Greetings and good health
Thank you very much for your valuable comments in the first round of Delphi, the study "**Nurses' educational needs in the oral health of inpatients**: **Delphi method** ". Researchers declare, your response in second round analyzed. At this round, please, announce your opinion on whether you agree with the items.

Thank you for patiently accompanying us in this study.

| ≠ | Oral health education priorities for nurses | agree | disagree |
| --- | --- | --- | --- |
| 1 | Oral Anatomy and physiology |  |  |
| 2 | Learning the signs and symptoms of common oral diseases |  |  |
| 3 | The relationship between diseases such as respiratory diseases, diabetes, heart disease, etc. and oral health problem |  |  |
| 4 | Training in managing dental emergencies (Sudden toothache, cheek swelling due to toothache, etc.). |  |  |
| 5 | Problems with the use of dentures and how to remove them |  |  |
| 6 | Leaning the oral medications and oral medication administration |  |  |
| 7 | Learning drugs that cause damage to the mouth and teeth |  |  |
| 8 | Familiarity with interventions for controlling halitosis (bad breath) |  |  |
| 9 | Patient education for tooth brushing and taking care of the mouth, especially in the elderly patient |  |  |
| 10 | Providing oral and dental care for unconscious patients |  |  |
| 11 | Providing oral and dental care for NPO patients |  |  |
| 12 | Providing oral and dental care for patients undergoing chemotherapy and radiotherapy |  |  |
| 13 | Providing oral and dental care for patients under oropharyngeal candidiasis |  |  |
| 14 | Providing oral and dental care for patients with maxillofacial trauma |  |  |
| 15 | Providing oral and dental care for patients admitted to critical care unit |  |  |
