# Supplementary material for: Nurses’ educational needs in the oral health of inpatients at Yazd Province in Iran: a Delphi study
Source: BMC Nurs. 2020 Dec 11;19:120. doi: 10.1186/s12912-020-00517-8 (PMC7733290; doi:10.1186/s12912-020-00517-8)
Supplement: Supplementary file 1 — Additional file 1. Delphi First Round Questionnaire [file 12912_2020_517_MOESM1_ESM.docx]

**Delphi First Round Questionnaire**

Dear Professor
Greetings and best wishes

The following questionnaire is related to the project " **Nurses' educational needs in the oral health of inpatients**: **Delphi method** ". This questionnaire is related to the first round of Delphi study. You will be asked to answer the following question in detail and carefully within 10 days. All of your answers will be kept confidential.

Age: ...... Year

Gender: Male □ Female □

Work Experience: ..... Year
Field of study: Nursing □ Dentistry □

Level of Education: Master's Degree □ PhD □ density doctorates□

**In your opinion, what are the educational priorities of nurses in the field of improving the oral health of hospitalized patients?**

- -.........................................................................................................................................................................................
- -.........................................................................................................................................................................................
- -.........................................................................................................................................................................................
- -.........................................................................................................................................................................................
- -.........................................................................................................................................................................................
- -.........................................................................................................................................................................................
- -.........................................................................................................................................................................................
- -.........................................................................................................................................................................................
- -.......................................................................................................................................................................................
- -.........................................................................................................................................................................................
- -.........................................................................................................................................................................................
- -.........................................................................................................................................................................................
